# Poly(ethylene Terephthalate) Carbon-Based Nanocomposites: A Crystallization and Molecular Orientation Study

**DOI:** 10.3390/polym12112626

**Published:** 2020-11-08

**Authors:** Vasiliki F. Alexiou, George N. Mathioudakis, Konstantinos S. Andrikopoulos, Amaia Soto Beobide, George A. Voyiatzis

**Affiliations:** 1 Foundation for Research & Technology-Hellas (FORTH), Institute of Chemical Engineering Sciences (ICE-HT), Stadiou Str., GR-265 04 Rio-Patras, Greece; valexiou@iceht.forth.gr (V.F.A.); mathioy@iceht.forth.gr (G.N.M.); candrik@iceht.forth.gr (K.S.A.); 2 Department of Physics, University of Patras, GR-265 00 Rio-Patras, Greece; 3 Department of Materials Science, University of Patras, GR-265 00 Rio-Patras, Greece

**Keywords:** poly(ethylene terephthalate), carbon nanotubes, composites, polarized Raman, orientation

## Abstract

Hybrid polymeric materials incorporating carbon nanostructures or inorganic constituents stand as a promising class of materials exhibiting distinct but also complementary features. Carbon nanotubes have been proposed as unique candidates for polymer reinforcement; however, sustained efforts are further needed in order to make full use of their potential. The final properties of the reinforced polymer are controlled in part by the morphology and the eventual molecular orientation of the polymer matrix. In the present study, multiwall carbon nanotubes (MWCNTs) were utilized in order to reinforce polyethylene terephthalate (PET) composites. The effect of CNTs on the crystallization and the orientation of the structurally hybridized polymeric material has been investigated from the perspective of assessing their impact on the final properties of a relevant nanocomposite product. Functionalized MWCNTs were used to achieve their optimal dispersion in the polymer matrix. The physical properties of the composites (i.e., crystallinity and orientation) were characterized via differential scanning calorimetry, X-ray diffraction, and polarized Raman microscopy. The addition of well-dispersed CNTs acted as a nucleation agent, increasing the crystallization of the polyethylene terephthalate matrix and differentiating the orientation of both CNTs and macromolecular chains.

## 1. Introduction

The term hybrid has become common; however, the difference between hybrid materials and composites is not so clear. Yamada et al. [1] defined hybrid materials as mixtures of two or more materials with new properties created by new electron orbitals formed between each material such as covalent bonds between polymers. More recently, Nanko et al. [2] proposed a criterion for hybrid materials from the point of view of the purpose of hybridization, classifying them into three categories: (1) structurally hybridized materials (composites), (2) materials hybridized in chemical bond, and (3) functionally hybridized materials. According to this classification, nanocomposites that are intended to have a nanometer-scale structure of mixing are a kind of structurally hybridized material.

Carbon nanotubes (CNTs) constitute a particular expression of nanomaterials being among the stiffest and strongest nanofibers known since Iijima’s relevant report in 1991 [3]. Their exceptional electronic, optical, mechanical, chemical properties, and high aspect ratio [4,5,6,7,8,9] make them attractive for many applications, with numerous relevant research works being published every year. CNTs have attracted great attention in the manufacturing of polymer nanocomposites being considered as ideal reinforcement fillers in order to produce stiff, strong, multifunctional products [10,11]. However, the advantage of employing CNTs in high-performance polymeric composites, taking advantage of their promising theoretically and experimentally supported extraordinary properties, is limited during processing, since it is very difficult to disperse them effectively within polymer matrices. CNTs easily form agglomerates or bundles, due to strong van der Waals forces that exist between the nanotubes, hindering their dispersion. In an effort to minimize the problem of dispersion, the chemical functionalization of the CNTs is often performed to promote good dispersion within polymer matrices [12,13,14]. During the fabrication of polymer/CNT composites with improved mechanical properties, four key areas need to be approached and understood: the CNTs’ pristine nature, the CNTs’ dispersion, the polymer–CNT interfacial interaction, and the orientation of the CNTs and polymer matrix molecules. The latter applies especially if the final application is the production of fibers or yarns.

Polyethylene terephthalate, PET, is one of the most commercial thermoplastic polymers in the world. Therefore, many studies have been dedicated to the investigation of PET nanocomposites containing nanocarbons as fillers for potentially improved properties [15,16,17,18,19,20].

The high drawability of PET has made this polymer one of the most important polymers in the textile industries. The long-term aim of this study lies in the development of lightweight and flexible smart protective textiles, which are designated for daily use, by high risk personnel. In the current study, multiwall carbon nanotubes, MWCNTs, with covalent attached reactive groups to their surface, such as carboxylic acid, were used to promote the effective dispersion of CNTs into the PET matrix. Interfacial interactions of the –COOH groups at the CNTs and the C=O groups in the PET macromolecular chains might be promoted through the type of hydrogen bonding [20]. The functionalization of CNTs is considered as an effective method to enhance their interfacial adhesion and consequent compatibility with the polymer matrix, increasing their dispersion therein. Melt blending is one of the most common methods used to disperse CNTs in a thermoplastic polymer matrix. In this context, the PET/CNT composites were fabricated via melt-compounding using a twin-screw extruder, which is a commercially widely used simple method for the production of nanocomposites on an industrial scale. The percentage of crystallinity in a hybrid/composite material is one of the most important characteristics that influences its physicochemical behavior. An enhancement of tensile modulus and toughness was also reported for isotactic PP/MWCNTs composites, which are fabricated by melt-blending and manufactured via overflow microinjection molding; such enhancement has been attributed to the increased crystallinity and larger number of shish-kebab structures [21]. Therefore, it is important to understand the influence of the CNTs on the crystallization of PET nanocomposites. In addition, the properties of the reinforced polymer are ultimately controlled in part by the morphology and the molecular orientation of the structurally hybridized polymeric material. It is critical to comprehend how CNTs may influence the crystallization parameters, morphology, and orientation during the processing of the polyester composite materials in order to correlate them with the mechanical properties of the final composite, especially from the perspective of the development of fibers or yarns. Most research articles on PET/CNT nanocomposites, trying to correlate increased crystallization from the incorporation of CNTs and their influence on the mechanical properties, make wide use of X-ray diffraction (XRD) and differential scanning calorimetry (DSC) [16,20,22,23]; the same applies in the present study. The novelty of our work relies on the use of polarized Raman spectra in combination with DSC and XRD measurements to gain further understanding of the orientation behavior and crystallization of the PET/CNT composites. Polarized Raman spectroscopy is an analytical method that readily provides detailed information on molecular structure and orientation. The relevant knowledge obtained from this technique is of both academic and industrial interest to reveal relationships between microstructure and macroscopic physical properties in polymers.

## 2. Materials and Methods 

### 2.1. Materials

The extrusion grade polyethylene terephthalate (PET) Relpet^®^ G5801 from Reliance Industries Limited, Maharashtra, India (IV 0.80 dl/g) was used. The carbon nanotubes were carboxyl functionalized multiwalled carbon nanotubes (Cheap Tubes Inc., Cambridgeport, MA, USA) CCVD grown, with a length of 10–20 μm, outer diameter of 30–50 nm, and 0.7% COOH groups. PET compounds with different wt % MWCNT loadings were produced in pellet form by D. SOURIS and Co S.A, Attiki, Greece, via a Leistritz ZSE 67 GG extruder. Polymer films having a thickness of about 150 μm were prepared by melt pressing the pellets at 290 °C and 30 bar followed by quenching in ice water; thus, low crystallinity films were obtained. Dog-bone-shaped samples were cut and stretched in a homemade stretching element [24]. The draw ratio, λ, is the ratio of the extended length to the original length determined from the displacement of ink marks on the filmstrip cut from the narrow midsection of the dumbbell-shaped test strip. 

### 2.2. Experimental Techniques

#### 2.2.1. Raman Measurements

The Raman spectra were recorded on a T-64000 (HORIBA Jobin Yvon, Edison, NJ, USA) micro-Raman system equipped with a 2D-CCD Symphony II detector. The excitation wavelength (514.5 nm) was provided by a DPSS laser (Cobolt Fandango TMISO laser, Norfolk, UK). The laser power on the sample was maintained at 1.3 mW and focused on the samples by a microscope objective 50× (NA = 0.55). The collected scattered beam passed through an appropriate edge filter for the removal of the strong elastically scattered photons (LP02-514RU-25, Laser 2000, Cambridgeshire, UK) and was directed into the slit of the monochromator in the single spectrograph configuration. The resolution was kept constant in all experiments (≈7 cm^−1^). The spectral range covered in the Raman measurements was ≈400–3200 cm^−1^. The polarized measurements were accomplished by using an appropriate rotator in the incident beam and a set of polarizer and broadband λ/2-plate in the scattered beam. The notation of Raman polarization measurements comprises a combination of three letters, such as *v*-VV. The small letter in italics (*v*) denotes the orientation of the draw axis relative to the laboratory-fixed coordinates. The two capital letters (HH or VV, H for horizontal and V for Vertical) denote the polarization direction of the excitation and scattered light on the measurement site with respect to the directions of the reference lab frame. In the present study, we define the polarization ratio R as the ratio of the intensities of a Raman peak in the parallel and the cross-polarization geometry with respect to the drawn stretching axis:

(1) $$R=\frac{I_{v-\mathrm{VV}}}{I_{v-\mathrm{HH}}}$$

Spectral calibration involved regular measurements of the Si reference sample or/and an Hg calibration lamp, while the system’s calibration with respect to the polarization response was achieved by a collection of a set of four spectra using all different polarization geometries from a CCl_4_ reference sample. 

#### 2.2.2. Differential Scanning Calorimetry

The crystallization and melting processes of net polymer and carbon-based nanohybrids were studied by DSC using a TA instruments Q100 thermal analyzer at a heating rate of 10 °C/min. All measurements were performed in a nitrogen atmosphere (50 mL/min). The specimens were heated from 25 to 300 °C and subsequently jump cooled to 25 °C, after which they were heated and cooled again. 

#### 2.2.3. X-ray Diffraction Measurements

The XRD spectra were performed for the structural characterization of the pure PET polymer and of the nanohybrids by using a Bruker D8 Advance diffractometer equipped by a Cu lamp (λCuKa = 1.54046 Å) at a scanning rate 0.02°/min over a range 2–60° (2θ).

## 3. Results and Discussion

### 3.1. Crystallization of PET/CNTs Composites

**Figure 1:**
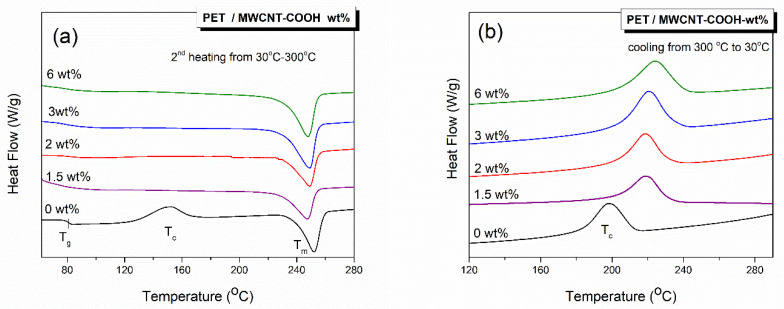
Differential scanning calorimetry (DSC) thermograms showing: (**a**) the second heating run, for neat polyethylene terephthalate (PET) and PET/multiwall carbon nanotubes (MWCNT) composites and (**b**) the crystallization exotherms from the melt/cooling cycle from 300 to 25 °C.

It is known that the information from the first heating cycle of the DSC refers to the actual state of the polymer crystals, and a subsequent cooling cycle erases the previous thermal history. Data obtained from the second heating cycle permit a direct comparison of the thermal properties of the polymer, since the thermal history was erased through the first heating cycle. Figure 1 shows the DSC thermograms of films of pure PET and nanocomposites with CNTs content between 1.5 and 6 wt % for the second cooling and heating run (after deletion of the thermal history). The glass transition temperature (*T*_g_), the cold crystallization temperature (*T*_cc_), the crystallization temperature from melting (*T*_c_), the melting temperature (*T*_m_), and the crystallization content (*X*_c_, %) were determined and are shown in Table 1. Subscript 2 indicates the second cycle. *X*_c_ % was calculated using:

(2) $$Xc=\frac{\Delta H_{m2}-\Delta H_{\mathrm{cc}2}}{\Delta H_{f}\;\left(1-w_{\mathrm{MWCNT}}\right)}$$

Δ*H*_m2_ is the enthalpy of melting and Δ*H*_c2_ is the absolute value of the enthalpy of cold crystallization, which are both determined by DSC. Δ*H*_f_ is the enthalpy of fusion of a completely crystalline material, and for PET, it is given as 140 J/g [25].

According to Figure 1a, in the thermogram of neat PET film(s), there is evidence of three thermal events: *T*_g_, *T*_cc_, and *T*_m_. However, upon the addition of MWCNTs, the cold crystallization peak of PET disappeared, indicating that the polymer was already crystalline. Clearly, the presence of MWCNTs enhances the crystallinity due to their nucleating effect. On the other hand, the addition of MWCNTs had little effect on *T*_g_, with a value of 81 °C for neat PET to 80 °C for the composite with 1.5 and 2 wt % MWCNT loadings. For compositions with higher MWCNT content, no *T*_g_ was detected, indicating that the PET chain dynamics are hindered [26,27]. There was also a change, albeit small, in the melting temperature, *T*_m_, with the addition of CNTs, from 252 °C in neat PET to ≈248 °C for all nanocomposites. 

In Figure 1b, the crystallization temperature of neat PET film was measured at 198 °C. It was increased to 218 °C after the addition of either 1.5 or 2 wt % and to 220 and 223 °C with the addition of 3 and 6 wt % MWCNTs, respectively. Subsequently, the degree of super cooling (Δ*T* = *T*_m2_ − *T*_c2_) decreased with the addition of MWCNTs. The increase in the *T_c_* and decrease in Δ*T* further confirms that the MWCNTs act as nucleating agents for PET crystallization; lower energy consumption is required for the crystal growth upon the incorporation of MWCNTs [27,28].

According to Table 1, the percentage of crystallinity indicates that the PET film was not fully amorphous, and the incorporation of MWCNT into PET enhanced the crystallinity degree. This again confirms that CNTs provide the nucleation sites for the PET from the melt to become more crystalline. These results are in quite good agreement with the Raman and XRD data shown below.

**Figure 2:**
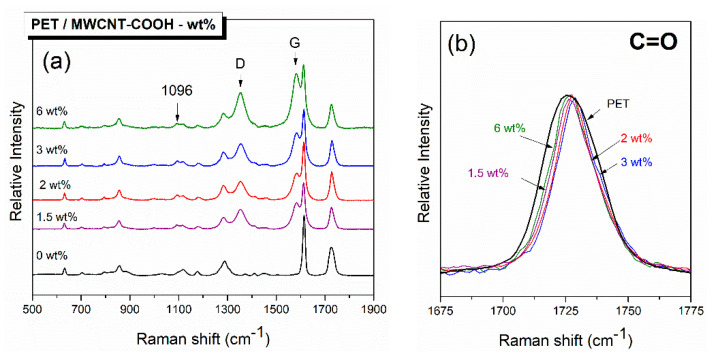
Raman spectra of neat PET and PET/MWCNT composites films (**a**) in the spectral region 500–1900 cm^−1^ and (**b**) in the vibrational window of the C=O stretching.

The collected Raman spectra from neat PET and PET/MWCNT composite films are depicted in Figure 2 in the spectral window from 500 to 1900 cm^−1^. The spectrum of PET displays the most characteristic vibrational peaks at 1616 cm^−1^, which are attributed to the symmetric stretch of the 1,4-para di-substituted benzene ring, and at 1725 cm^−1^, corresponding to the carbonyl stretching. As MWCNTs are added, their characteristic peaks located at 1350 and 1580 cm^−1^ (marked with arrows) are easily observed even though there is partial overlapping with PET bands; they are attributed to the *D* and *G* band, respectively [29,30,31]. The intensity of these peaks due to the presence of MWCNTs increases with the increasing carbon nanotube content. 

Analysis of the Raman spectra collected from the composite samples depicted in Figure 2 indicates that upon the addition of MWCNTs to PET, a new peak emerged at 1096 cm^−1^, while the C=O stretching band got sharper (Figure 2b). According to the literature, [32,33,34,35,36] crystallization induces changes in the Raman spectrum of PET. Spectral features appear in crystalline samples at ≈1096 cm^−1^ that are absent in the amorphous one. This band (at 1096 cm^−1^) represents a combination of C–O stretching, COC bending, CCO bending, and C–C stretching in the ethylene glycol segment. It is noteworthy that the crystallization is also correlated with the width of the carbonyl band at ≈1725 cm^−1^, which becomes sharper. The C=O stretching band is a combination of three bands, the ones at 1721 and 1735 cm^−1^ are correlated with the amorphous state, and the band at 1726 cm^−1^ corresponds to the crystalline state. In Figure 2b, the addition of CNTs decreases the width of the C=O stretching band, indicating an enhancement of the crystalline counterpart at the expense of the other two. It is also perceptible that for the composite with 6 wt % MWCNT loading, the bandwidth of the carbonyl vibration widens, and the intensity of the peak at 1096 cm^−1^ slightly decreases with respect to the composite with 3% CNTs loading. The crystallinity of PET increases with the addition of MWCNTs (increases the number of nucleation sites), but it begins to fall slightly as more MWCNTs are added, which is most probably due to the difficulties in dispersing them effectively in the polymer at higher loadings. 

**Figure 3:**
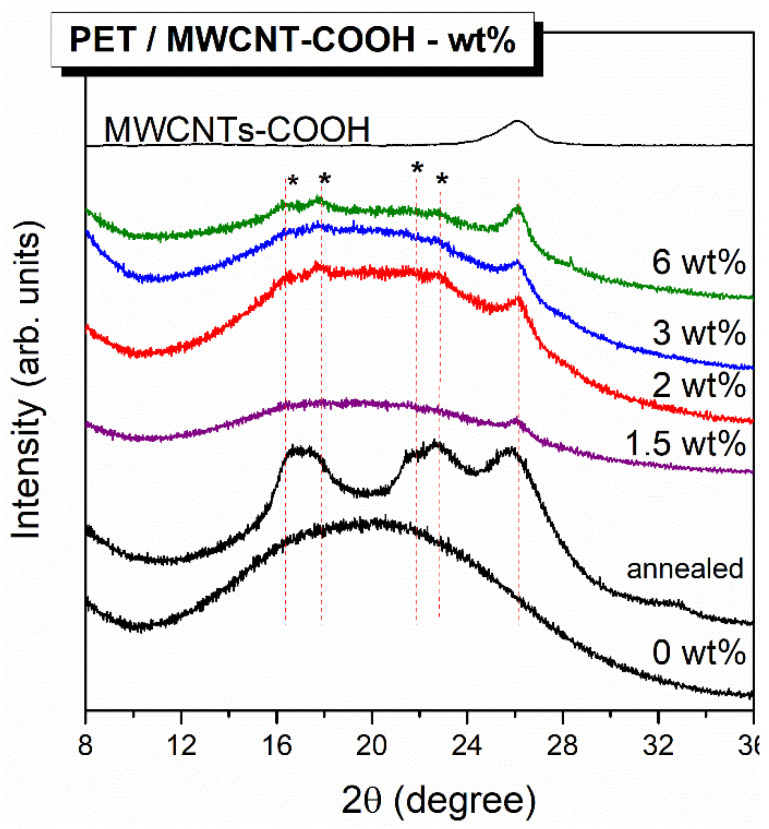
XRD profiles of neat PET and PET/MWCNT composites films.

X-Ray diffraction (XRD) is very well suited for the study of partially crystalline materials; therefore, it was applied for the estimation of crystallinity of PET/CNTs composites in film form. Figure 3 presents the XRD patterns of films of pure PET and PET nanocomposites with CNTs content between 1.5 and 6 wt %; the diffraction peak of MWCNT-COOH at 2θ = 26° labeled as d(002), which is in good agreement with previous studies [37], is also shown for comparison. Similarly, the diffraction patterns for an annealed PET sample, bearing a crystallinity of 25% as determined by DSC thermograph, is also depicted for comparison. An amorphous diffraction broad peak was perceived for PET neat film; however, specific X-ray peaks were even marginally detectable for samples loaded with CNTs. Crystal faces for highly crystalline PET have been assigned in the literature to the following diffraction peaks at 2θ degrees: (0–11) = 16.3 °, (010) = 17.5°, (–111) = 21.5°, (110) = 22.7°, (100) = 26.1°, and (1–11) = 27.65° [22,38,39]. Similar diffraction peaks are shown to be developed with increasing MWCNT loading due to the already assigned nucleating effect of CNTs that promotes PET crystallization. More specifically, the peaks corresponding to crystalline PET at 16.3°, 17.5°, 21.5°, and 22.7° are detectable, though hardly; however, the peaks at 26.1° and 27.65° are masked by the intense CNTs refraction peak at 26°. The latter is clearly detectable in all the PET/CNTs compositions

### 3.2. Molecular Orientation

**Figure 4:**
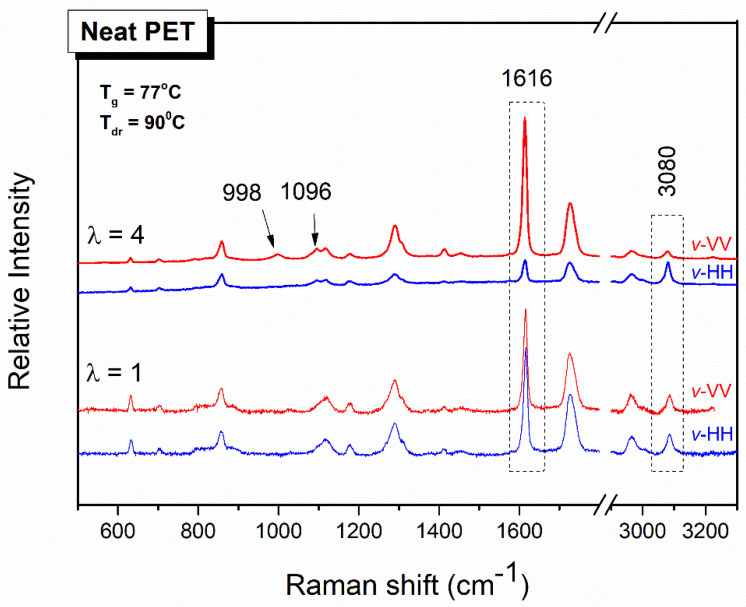
Polarized Raman spectra of PET film undrawn, λ = 1, and drawn to λ = 4 in two different polarization geometries, VV and HH, with respect to the position of the specimen relative to the laboratory-fixed coordinates, *v*. The spectra are shifted along the intensity axis for clarity. H: Horizontal, V: Vertical.

The dog-bone-shaped test strips cut from the PET/MWCNT composite films were uniaxially stretched at 85 °C (10 °C above the *T*_g_) and at different draw ratios, λ. In Figure 4, the typical polarized Raman spectra of a representative PET film before and after uniaxial drawing are depicted, using two polarization geometries. The 1616 cm^−1^ peak of PET, as already mentioned, corresponds to the Raman activity of the symmetric stretch of the skeletal 1,4-para substituted benzene rings. Before drawing, there is no preferred orientation, since the sample is essentially isotropic at molecular level; thus, no differences were found between VV and HH scattering intensities for all spectral features. However, when the sample is stretched, e.g., up to a draw ratio λ = 4, the VV and HH spectra develop differences in relative band intensities. The extent of such differences depends on the position of the sample, *v*-vertical, with respect to the laboratory-fixed coordinates, and it reflects the anisotropy induced by the drawing process. For the drawn sample, the scattering intensities of the skeletal vibrational modes (for example, 1616 cm^−1^) are higher in parallel to the draw direction polarization geometries, *v*-VV, than in the corresponding cross-polarization geometries, *v*-HH. That means that there are more scatterers of para-di-substituted benzene rings in the *v*-VV geometries, more benzene rings aligned toward the draw direction, and more macromolecular chains parallel to the draw direction. However, if we pay attention to the peak at 3080 cm^−1^, which is attributed to CH stretching of the benzene ring, it is evident that its behavior is opposed to that of the 1616 cm^−1^ peak. That is, the intensity of the 3080 cm^−1^ peak is favored in the perpendicular to the draw direction polarization geometry, indicating that this vibration is perpendicular to the skeletal macromolecular chain, as already invoked before [40].

**Figure 5:**
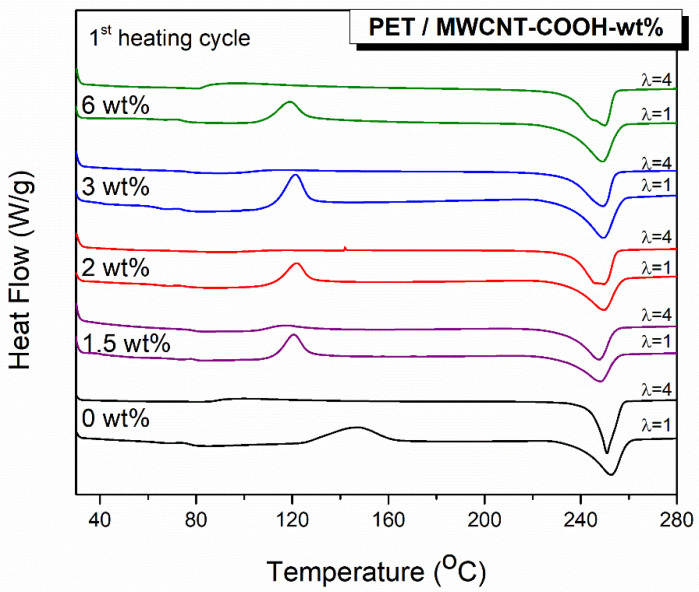
DSC thermograms for unstretched and stretched neat PET and PET/MWCNT composites films. The results presented correspond to the first heating cycle, where the information refers to the actual state of the polymer crystals.

Two other scattering features are generated in the Raman spectra of PET films after stretching, at 1096 and 998 cm^−1^, as indicated with arrows in Figure 4. As mentioned above, the band at 1096 cm^−1^ has been correlated to the crystallinity of PET. The band at 998 cm^−1^ is related to the existence of *trans* conformation in the chain axis [34,41]. These two peaks are not observable in the unstretched almost amorphous PET film. The PET film stretched to λ = 4 generates Raman bands assigned to crystallinity, indicating stretch-induced crystallization. These results are in excellent agreement with the DSC data shown in Figure 5 below. There, the cold crystallization peak of the unstretched PET film appears at 151 °C but is absent for the stretched one. This indicates that the polymer chains undergo stretch-induced crystallization; no further crystallization occurs during the DSC thermograph, as is the case for the unstretched PET film. The melting temperature for both films appears near 252 °C, with the melting peak of the stretched film being sharper, indicating a more ordered oriented-induced crystallization with a narrow crystallite size distribution. The significant increase in the crystallinity from the unstretched to the stretched film from 7 to 24.2% was attributed to the orientation-induced crystallization upon stretching. Similarly, the thermograms of the 1^st^ heating cycle for the stretched PET/MWCNT composite films do not exhibit an exothermic *T*_c_ peak, indicating that the polymer chains are well crystallized as a consequence of the stretching. However, differences are found for the melting endothermic peaks when comparing stretched net PET with PET/MWCNT composite films. As mentioned above, the melting peak for the neat stretched PET film is narrower that the one obtained for the unstretched film, suggesting more ordered and perfect crystallites. When MWCNTs have been incorporated into PET, the melting endothermic peak of the stretched composite films at ≈250 °C is not narrower than the unstretched ones, but on the contrary, it is wider and asymmetric; a second peak at lower temperatures, ≈245 °C, is easily noticeable for almost all PET/MWCNTs compositions. These results indicate that the crystalline content is derived from a combination of the addition of MWCNTs acting as nucleating agents together with strain-induced crystallization. Table 2 gathers the values of the glass transition temperature, crystallization temperature, melting temperature, and crystallization content for unstretched and stretched PET/MWCNT composite films evaluated from the first heating run where information refers to the actual state of the polymer crystals. 

**Figure 6:**
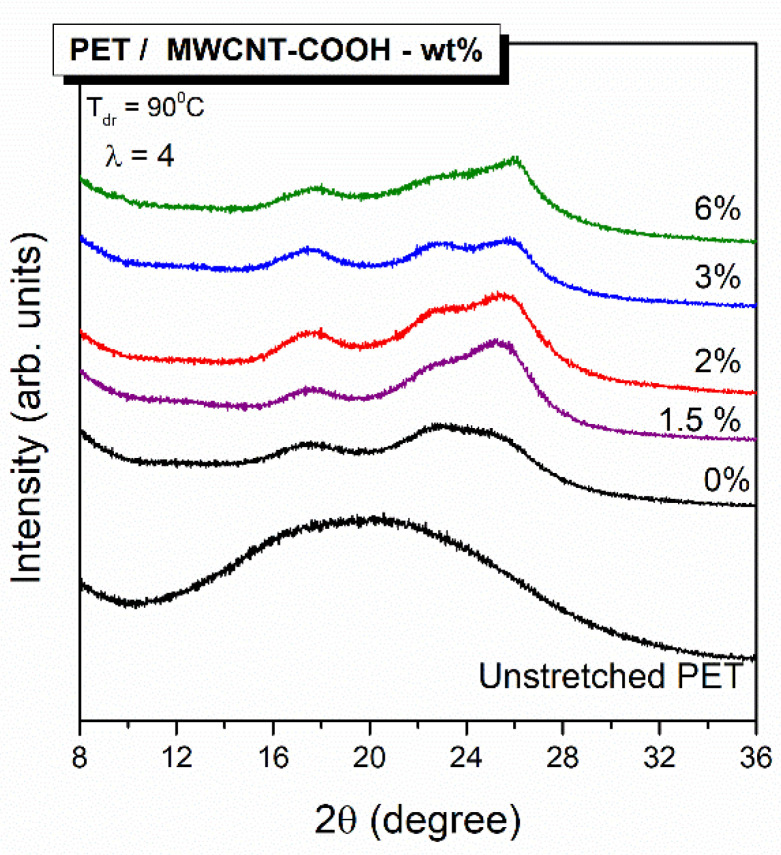
XRD profiles of neat PET and PET/MWCNT composites films uniaxial stretched to draw ratio λ = 4. The XRD pattern of an unstretched PET film is depicted at the bottom for comparison.

Uniaxially oriented films of neat PET and PET nanocomposites incorporating 1.5, 2, 3, and 6 wt % MWCNTs were also measured by XRD, and the resulting patterns are depicted in Figure 6; the XRD pattern of an unstretched PET film is also presented for comparison. Three main characteristic crystal faces of PET are evident for all stretched polymer films positioned at 17.5°, 22.7°, and 26° values of 2θ corresponding to (010), (-110), and (100) crystal planes, respectively. It is noteworthy that for the samples containing CNTs, the (100) crystal plane at 26° is more pronounced, and this is a consequence of the contribution of the (002) crystal face corresponding to CNTs placed at 2θ = 26°. The XRD measurements confirm that composite samples containing CNTs showed orientation-induced crystallization. In addition, we could argue that this stretch-induced crystallization is favorable for PET/MWCNT samples with a lower than 6 wt % loading in CNTs, since the crystal planes are more intense for these composite films.

**Figure 7:**
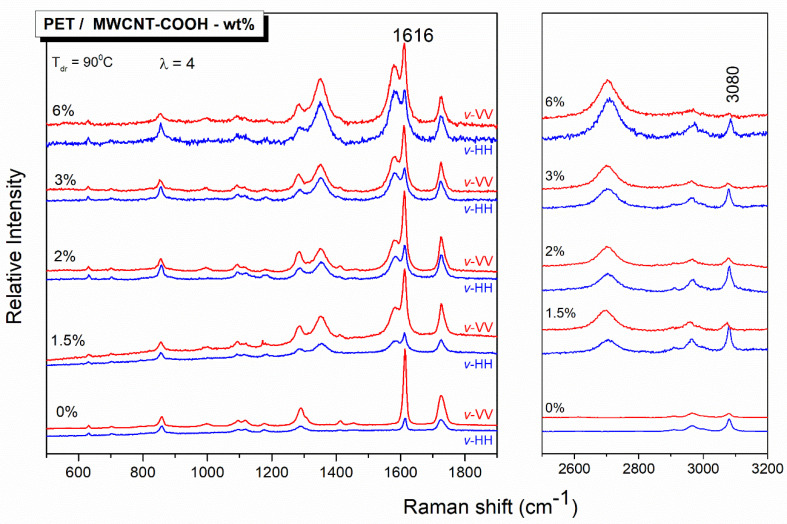
Polarized Raman spectra of PET/MWCNT films, with different carbon nanotube content, drawn to λ = 4 in two different polarization geometries, VV and HH, with respect to the position of the specimen relative to the laboratory-fixed coordinates, *v*.

Polarized Raman spectroscopy was also applied to study the PET–MWCNTs nanocomposites; the results are depicted in Figure 7 for films stretched to a draw ratio of 4. The CNTs vibrational contribution at 1350 (D band) and 1580 cm^−1^ (G band) partially overlaps with PET bands; their G’ counterpart is also observed at around 2700 cm^−1^. For all PET/MWCNTs compositions, the intensity of the PET skeletal vibrations, similar to that at 1616 cm^-1^, exhibits higher intensity parallel to the draw axis polarization geometry, *v*-VV, than that in the cross-polarization geometry, *v*-HH. In what refers to the carbon nanotubes, the intensities of both D and G bands are also higher parallel to the draw axis polarization geometry. The G’ band does not seem to be sensitive to the orientation [17]. Another interesting point is the fact that the polarization ratio R (*I_v-_*_VV_/*I_v-_*_HH_) of the 1616 cm^−1^ skeletal PET band decreases by increasing the carbon nanotube content in the PET matrix. In other words, with a similar reasoning, it seems that the molecular orientation of PET macro chains decreases with the increase of the CNTs loading in PET–MWCNTs nanocomposites. 

The average R (*I_v-_*_VV_/*I_v-_*_HH_) values (over a number of ten statistical measurements from different μm size spots on the samples) for three selected vibrational modes together with the respective standard deviations are summarized in Table 3.

The 1616 cm^−1^ band, assigned to the symmetric stretching of the 1,4-carbons of the benzene ring, represents the orientation of the backbone polymer chain, while the peak at 3080 cm^−1^, which is related to the ring C-H stretching, evaluates the orientation of species that tend to orient perpendicular to the polymer chain. Finally, the 1580 cm^−1^ band describes the orientation of the CNTs. For skeletal vibrational modes (e.g., 1580 and 1616 cm^−1^), R takes values >1 and increases with increasing molecular orientation, whereas R values < 1 are obtained for vibrational modes associated with species that tend to orient perpendicular to the chain axis (e.g., 3080 cm^−1^). For the latter case, the R-values tend to diminish with the increase of anisotropy. 

**Figure 8:**
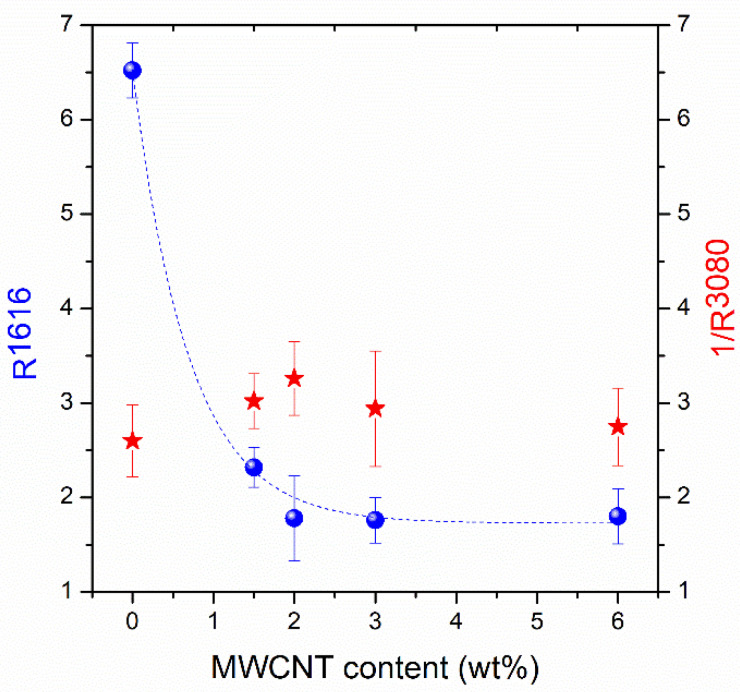
R values for the 1616 cm^−1^ band and 1/R values for the 3080 cm^−1^ band as a function of MWCNTs content.

The R-values for the 1616 cm^−1^ band and the 1/R-values for the 3080 cm^−1^ band are plotted as a function of CNT loading in Figure 8 for samples stretched to λ = 4. The higher the CNT content, the lower the *R^1616^* values, indicating that the addition of CNTs results in a loss of anisotropy of the PET matrix. A study performed by Mazinani et al. [17] has reported that the addition of CNT in PET melt-spun fibers decreases the degree of orientation due to the modification of flow conditions around the nanoparticles added to the polymer matrix. The applied elongation field on the pure polymer phase is reduced by converting a fraction of the elongation flow into a shear component at the CNT/polymer interphase. This reduction in the strength of the elongational flow imposed on the polymer melt was proposed in order to explain the restriction of the overall degree of orientation in CNT nanocomposites compared to the pure PET fibers. Similar results have been also previously observed in PET/nanoclay fibers and PET/carbon nanotube nanofibers [17,42]. Nevertheless, the explanation of the reduction of *R^1616^* values in Figure 8 is not straightforward. If a loss of segmental orientation in PET macromolecular chains in their CNT composites was the case, the *R^3080^* values as a function of wt % MWCNT should increase. The results summarized in Table 3 indicate that this is not the case; i.e., the trend for the *R^3080^* values is to decrease with CNT concentration. This behavior can be seen in Figure 8, where 1/*R^3080^* is plotted vs. wt % MWCNTs and suggests an increase of orientation with the addition of MWCNTs, in contrast to the results obtained from the orientation evaluation through the 1616 cm^−1^ band. The controversy can be explained by taking into account the orientation of CNTs in the polymer matrix. Zhang et al. [43] have shown that the absorption of visible light of SWCNTs depends on light polarization. Hence, the absorption of light from an individual CNT is higher when the light polarization is along its axis of anisotropy and is minimal when the polarization of light is perpendicular. Zhang et al. demonstrated this by a visual inspection of ice melting in the favorable geometry in contrast to the non-melting observed in the alternate geometry. In order to explain the results shown in Figure 8, someone should take into consideration not only the segmental orientation of the PET chains but also the absorption phenomena induced by the presence of MWCNTs in the polymer matrix; evidence of such phenomena in the anisotropic composites is given by the transmission of visible polarized light through them, which is considerably suppressed when the polarization used is parallel to the axis of anisotropy (Figure S1). In this concept, the Raman band intensities in spectra using the *v*-VV geometry suffer from intensity loss due to increased absorption from the CNTs, while the respective intensities in the *v*-HH geometry experience weaker absorption. In principle, this is the reason that the trend of the *R^1616^* and 1/*R^3080^* values was found to decrease and increase, respectively. It has to be stressed that thermally induced orientation relaxation caused by the illumination of the highly absorbing (due to the MWCNTs) samples should be neglected, at least for the low/moderate laser powers used for the excitation. Figure S2 demonstrates that the R^1616^ values extracted for the different laser powers on the samples are the same (within experimental error). 

Conversely, the phenomena of segmental orientation alteration due to the interaction of PET segments with the MWCNTs should not be ignored; however, their evaluation through polarized Raman spectroscopy is particularly difficult due to the absorption phenomena described above. An interesting finding that may be associated with the MWCNTs effect on PET orientation is that the wt % dependence of the R^1616^ and 1/R^3080^ values is not monotonic. Such effects may be understood in the concept of the formation of alternate shish-kebab type crystal structures in the vicinity of the CNTs reducing orientation [44] or/and the eventual influence of the CNTs on the occurrence of mesophase nucleation prior to crystallization [45]. In any case, the present study aimed to take a further look at the complexity of CNT-induced morphology development of polymers during uniaxial deformation. The knowledge obtained will support a future work dealing with relevant fibers/yarns. The eventual additional orientation of both polymer chains and CNTs as well as the stretch-induced crystallization anticipated for the polymer chains will be of great interest. In this case, wide-angle X-ray diffraction (WADX) measurements would contribute to clearly elucidate molecular orientation, even for complicate crystalline structures such as shish-kebab. 

## 4. Conclusions

Differential scanning calorimetry, X-ray diffraction, and polarized Raman spectroscopy have been utilized for the investigation of the crystallization, molecular structure, and the orientation of the structurally hybridized PET/MWCNT polymeric materials. Well-dispersed CNTs acted as a nucleation agent, increasing the crystallization of the polyethylene terephthalate matrix. This was confirmed by the DSC measurements and reflected in the XRD patterns with diffraction peaks at the same 2θ angles than the ones associated with crystalline PET crystal faces and revealed by Raman vibrational features correlated to PET crystallinity. Uniaxial stretching of the PET/MWCNT composite films was accompanied by an increase of the crystallinity attributed to the stretch-induced crystallization, which was confirmed by DSC, XRD, and Raman measurements. Molecular orientation of PET macro chains, by means of polarized Raman spectroscopy, appears to decrease with increasing CNTs loading in PET–MWCNTs nanocomposites. However, the evaluation of this macromolecular orientation is particularly difficult due to light absorption phenomena from the presence of potentially oriented CNTs. There is evidence of complexity of the CNT-induced morphological development of polymers during uniaxial deformation.

## Figures and Tables

**Table 1:** Effect of MWCNT addition on thermal parameters for PET and PET/MWCNTs composites.

Sample	*T*_g2_ (°C)	*T*_cc*2*_ (°C)	*T*_m2_ (°C)	*T*_c2_ (°C)	%*X*c	Δ*T* (°C)
PET	81	151	252	198	10	54
PET/MWCNT -1.5 wt %	80	-	248	218	24.7	30
PET/MWCNT -2 wt %	80	-	249	218	23.6	31
PET/MWCNT -3 wt %	-	-	249	220	36.4	29
PET/MWCNT -6 wt %	-	-	248	223	36.3	25

**Table 2:** Effect of stretching on thermal parameters for PET and PET/MWCNTs composites. Subscript 1 indicates the first heating cycle.

Sample		*T*_g1_ (°C)	*T*_cc1_ (°C)	*T*_m1_ (°C)	%*X*c
PET	λ = 1	78	147	252	7.2
	λ = 4	-	-	250	24.2
PET/MWCNT -1.5 wt %	λ = 1	78	121	248	12.8
	λ = 4	78.5	117	(245)/248	23
PET/MWCNT -2 wt %	λ = 1	75	121	250	15.5
	λ = 4	82	-	245/250	30
PET/MWCNT -3 wt %	λ = 1	74	121	250	17.7
	λ = 4	76	-	245/250	31.4
PET/MWCNT -6 wt %	λ = 1	74	119	249	23
	λ = 4	78	-	244/250	32.5

**Table 3:** Polarization ratio values obtained from polarized Raman spectroscopy measurements for PET and PET/MWCNTs composite films stretched to a draw ratio of 4.

Sample	*R^1616^*	*R^1580^*	*R^3080^*	1/*R^3080^*
PET	6.52 (± 0.29)	-	0.385	2.60 (± 0.38)
PET/MWCNT -1.5 wt %	2.32 (± 0.21)	1.37 (± 0.08)	0.331	3.02 (± 0.29)
PET/MWCNT -2 wt %	1.78 (± 0.45)	1.45 (± 0.09)	0.307	3.26 (± 0.39)
PET/MWCNT -3 wt %	1.76 (± 0.24)	1.17 (± 0.14)	0.340	2.94 (± 0.61)
PET/MWCNT -6 wt %	1.80 (± 0.29)	1.35 (± 0.08)	0.364	2.75 (± 0.41)

## Supplementary Materials

The supplementary materials are available online at https://www.mdpi.com/2073-4360/12/11/2626/s1.
