# Supplementary material for: Poly(ethylene Terephthalate) Carbon-Based Nanocomposites: A Crystallization and Molecular Orientation Study
Source: Polymers (Basel). 2020 Nov 8;12(11):2626. doi: 10.3390/polym12112626 (PMC7695265; doi:10.3390/polym12112626)
Supplement: Supplementary file 1 [file polymers-12-02626-s001.pdf]

Supplementary Information

Poly(ethylene terephthalate) carbon-based nanocomposites: a crystallization and molecular orientation study

In order to check the anisotropy in the optical properties exhibited by the uniaxially stretched pristine and composite film, the transmission of visible light possessing selected polarization through the corresponding films was checked using the 514.5 nm wavelength of a DPSS laser. More specifically the uniaxially stretched PET and PET-1.5%MWCNTs films (λ=4 for both films) were placed between the laser and a single channel detector with their anisotropy axis in the horizontal (*h*) position. The detector measured the power (in mW) of the transmitted beam in two specifically selected polarization geometries: (a) vertical (V) which is the polarization of the laser beam and (b) horizontal (H) achieved by placing an appropriate light polarization rotator at laser exit. The ratio of the transmitted laser power in the *h*-V and *h*-H configuration ($T^{h\text{-}V}/T^{h\text{-}H}$) was calculated for three measurements performed at different spots on the sample and is plotted in Fig. S1 (the throughput of the rotator is taken into consideration for the extraction of the values). For the isotropic film $T^{h\text{-}V}/T^{h\text{-}H}$ =1 while for the uniaxially stretched films $T^{h\text{-}V}/T^{h\text{-}H}$ >1. The considerably higher values exhibited by the composite is a result of the enhanced anisotropy in the absorption of aligned MWCNTs.

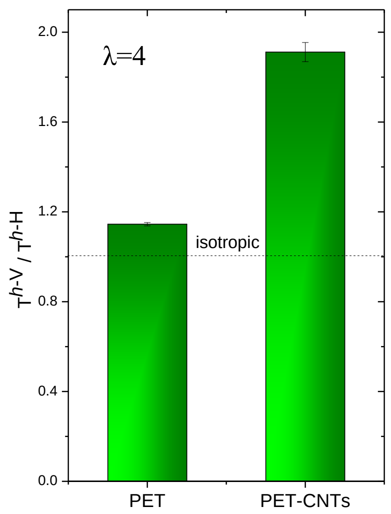

Figure S1. Ratio of the transmission (514.5 nm) through uniaxially drawn (λ=4) neat PET and PET-0.5 wt% MWCNTs specimens using the *h*-V and *h*-H configuration

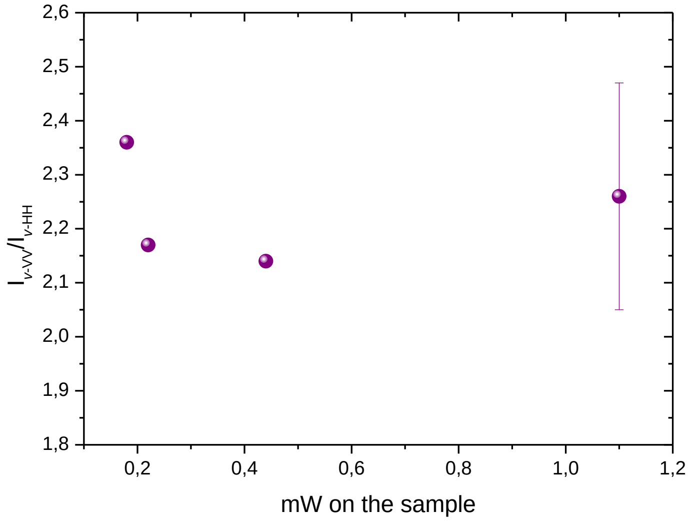

Figure S2. The polarization ratio R ($I_{v\text{-}VV}$ / $I_{v\text{-}HH}$) of the 1616 cm^{-1} skeletal band collected from the uniaxially drawn (λ=4) PET-1.5 wt% MWCNTs sample with different laser power intensities (0.18, 0.22, 0.44 and 1.1 mW) on sample and with the same collection time (25 min).
